# Experimental atherosclerosis models in small animals (Review)

**DOI:** 10.3892/mi.2026.324

**Published:** 2026-05-18

**Authors:** Mehmet Ertugrul Balkar, Hakan Parlakpinar

**Affiliations:** Department of Medical Pharmacology, Faculty of Medicine, Inonu University, 44280 Malatya, Türkiye

**Keywords:** atherosclerosis, experimental models, animal, endothelium, hyperlipidemia

## Abstract

Atherosclerosis refers to the accumulation of plaque in arteries and is the underlying cause of various serious vascular diseases, including coronary artery disease and ischemic stroke. These diseases pose a major threat to global health and are often fatal. Individuals predisposed to metabolic health conditions, such as familial hypercholesterolemia, hypertension or diabetes mellitus, often manifest atherosclerosis in the early stages. Because early-stage atherosclerosis is asymptomatic and typically progresses without obvious symptoms, its clinical detection remains challenging before the onset of notable vascular obstruction. The primary objective of the present review was to comprehensively address experimental atherosclerosis models that are commonly used in small animals, and to serve as a source of inspiration for the development of new pharmacological treatment protocols in the future. Numerous sources related to the importance of atherosclerosis, the disease process, pathophysiology, risk factors and experimental models developed in small animals were examined for the present review. Experimental atherosclerosis models are crucial for researchers to understand the disease process and to develop innovative treatments, and *in vivo* models reveal biochemical and molecular processes associated with the disease. Understanding the unique characteristics of each animal species is essential for selecting the appropriate experimental model, and creation of *in vivo* models requires careful consideration of animal selection, materials and model characteristics. Further research is needed to understand the pathophysiological processes of atherosclerosis and to develop novel pharmacological treatments for the diseases it causes.

## 1. Introduction

Atherosclerosis is a chronic inflammatory condition of large- and medium-sized arteries that causes ischemic heart disease, stroke and peripheral vascular disease, which are generally termed cardiovascular diseases (CVDs) (1). CVD is the most frequent cause of mortality and morbidity worldwide, with a large share of this burden borne by low- and middle-income societies (2). Atherosclerosis, which is the main pathological process underlying the majority of CVDs, is a mostly asymptomatic process, and thus, it is challenging to accurately assess its incidence (3).

Atherosclerotic plaque forms when arterial damage occurs; the resulting plaque can narrow or block blood flow and may cause local thromboembolism. Plaques are most often located in the aorta, coronary, cerebral and lower limb arteries (4). Notably, atherosclerosis is a major challenge to modern medicine, as it has a long developmental history, is prevalent worldwide and occurs in aging populations. Current management strategies have not been effective in substantially reducing risk factors, such as hypertension and diabetes (5). While clinical diagnosis of atherosclerosis primarily relies on advanced imaging techniques, such as angiography, vascular Doppler ultrasound and computed tomography, to evaluate vessel stenosis, these methods often detect the disease only after notable structural changes have occurred. Similarly, although therapeutic interventions involving lipid-lowering statins and anti-platelet agents focus on stabilizing existing plaques to prevent acute events, they offer limited insight into the early molecular triggers of atherogenesis. Because clinical trials in humans are constrained by ethical boundaries and the long duration of plaque progression, transitioning to experimental animal models becomes indispensable. These models allow for the detailed exploration of molecular pathogenesis and the testing of novel pharmacological strategies within a controlled and accelerated timeframe, bridging the gap that clinical observation alone cannot fill. Furthermore, the employment of animal models offers a distinctive opportunity to utilize a variety of induction methods, ranging from metabolic challenges and genetic modifications to controlled mechanical interventions, such as surgical denudation or minimally invasive percutaneous techniques, to replicate specific stages of human plaque development.

## 2. Pathophysiology and steps of atherogenesis

Arteries are structurally classified into two types: Elastic and muscular, and their walls consist of three layers: The tunica intima, tunica media and tunica adventitia. The innermost intima contains endothelial cells, whereas the media consists of smooth muscle cells that regulate vascular tone and composition of the extracellular matrix. The adventitia, the outermost layer of the vessel, consists of loose connective tissue containing fibroblasts, nerves and the vasa vasorum structures that supply large blood vessels (6,7). Atherogenesis, the complex process of plaque formation, is initiated by the accumulation of lipoproteins containing apolipoprotein (Apo)B in the subendothelial space. This process progresses through endothelial activation and immune cell involvement. The pathogenesis of this condition is characterized by the accumulation of cells, lipids and extracellular matrix components within the vascular wall, progressing through distinct stages of fatty streaks, fibrous plaques, and ultimately, advanced lesions (8-11). Dysfunctional endothelial cells, which are critical to vascular homeostasis, serve a fundamental role in initiating the atherosclerotic process by triggering the migration of inflammatory cells to lesion sites. During this process, monocytes that have migrated into the subintimal space differentiate into macrophages; these macrophages subsequently transform into lipid-laden foam cells by taking up oxidized low-density lipoprotein (ox-LDL) via scavenger receptors (12-14). Laminar shear stress has been shown to promote nitric oxide (NO) production and the expression of anti-atherogenic genes, thereby contributing to vascular health. By contrast, oscillatory shear stress in bifurcation regions has been observed to trigger endothelial dysfunction by increasing the expression of adhesion molecules. This biomechanical change has been shown to accelerate plaque development by promoting the adhesion of monocytes to the vascular wall, and the migration and proliferation of smooth muscle cells into the subintimal space (3,13,15). The proliferation of smooth muscle cells migrating into the subintimal space, leading to the formation of a fibrous cap and intimal hyperplasia, combined with the continuous recruitment of immune cells to the lesion site due to cytokines (such as tumor necrosis factor and interleukin-6) released during the chronic inflammatory process, results in plaque growth and hemodynamically important stenosis (16,17). In addition, the accumulation of ox-LDL in the plaque microenvironment has been shown to trigger the differentiation of macrophages into distinct inflammatory subtypes and the formation of foam cells with varying phagocytic capacities. This process leads to the development of a necrotic core through cell death and the progression of the plaque (18-20). The disruption of the intimal structure by necrotic cores within the plaque is a hallmark of this condition. Hemorrhages resulting from fragile neovascularization originating from the adventitial vasa vasorum and thinning of the fibrous cap due to chronic inflammation further compromise the stability of the plaque, increasing the risk of rupture and thrombosis (21,22).

Observing these intricate cellular and mechanical processes in an *in vivo* setting depends on the association of pathophysiological stages with particular experimental models. For example, early-stage processes, such as impaired lipid metabolism and foam cell accumulation, are simulated using accelerated dietary protocols in ApoE^-/-^ or LDL receptor (LDLr)^-/-^ transgenic models (23,24). In subsequent stages of the process, such as fibrous cap formation and necrotic core expansion, ‘humanized’ models that demonstrate human-like LDL aggregation are employed to enhance the clinical validity of the study. Humanized models demonstrating human-like LDL aggregation are primarily engineered to bypass the inherent atheroresistance of rodents by aligning their lipid profiles with human pathophysiology. The platforms under discussion refer to humanized models, including LDLr^-/-^ humanized ApoB100 (hApoB100) transgenic mice, which were generated by crossbreeding total LDLr^-/-^ strains with transgenic lines expressing hApoB100. This genetic strategy ensures that hApoB100 becomes the primary cholesterol carrier within the LDL fraction, thereby effectively replicating the hallmark characteristics of familial hypercholesterolemia (FH) (25). Additionally, somatic gene transfer using adeno-associated virus (AAV) vectors to deliver gain-of-function proprotein convertase subtilisin/kexin type 9 (PCSK9) mutations has emerged as a rapid method to induce severe hypercholesterolemia by triggering LDLr degradation without the need for extensive crossbreeding (24). The incorporation of human-specific genetic and viral components into these models enables the precise observation of subendothelial LDL entrapment and conformational changes to ApoB100 under clinically relevant conditions (26). Furthermore, critical complications such as fibrous cap thinning and rupture, which are rarely observed in standard mouse models, can be modeled using aged mouse groups or specialized genetic platforms containing the fibrillin-1 (Fbn1) mutation, which disrupts elastin integrity (27).

## 3. Risk factors, CVD progression and symptoms

**Figure 1:**
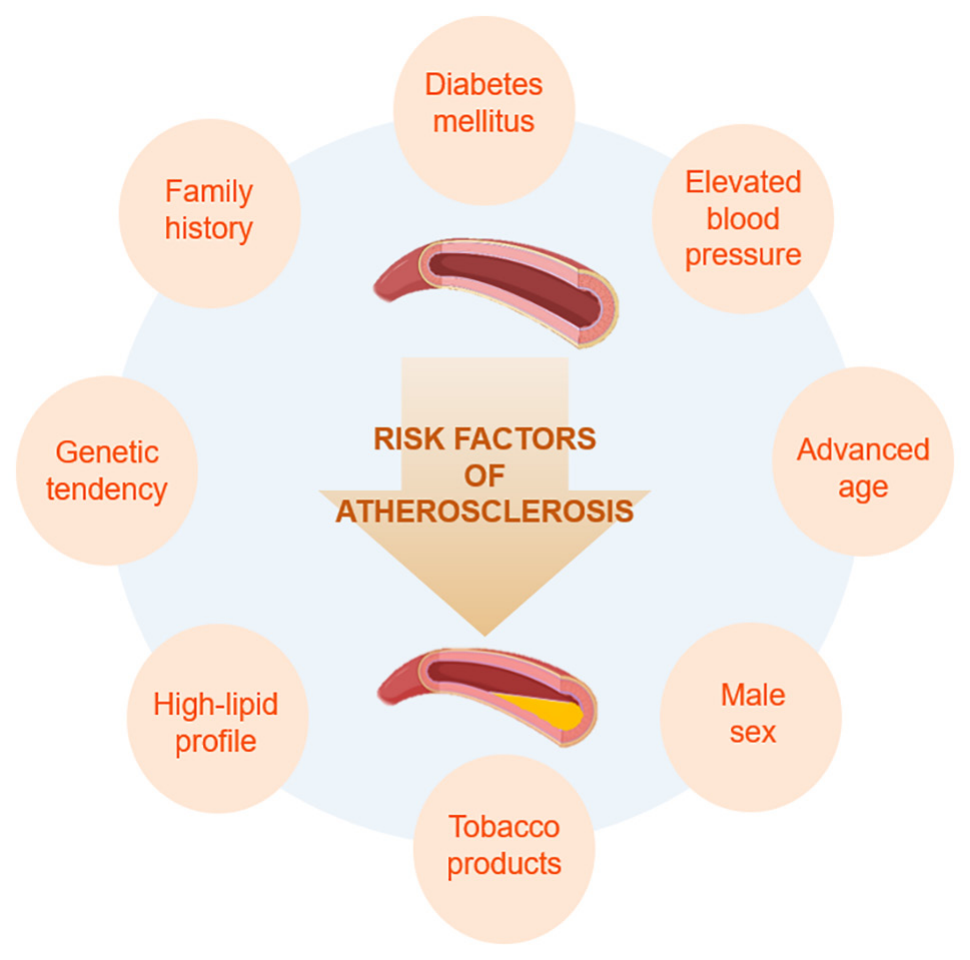
Risk factors of atherosclerosis.

The pathogenesis of atherosclerosis is a chronic inflammatory process shaped by the complex interactions between genetic predisposition and environmental triggers. Modifiable and non-modifiable risk factors serve a pivotal role in the disruption of vascular endothelial integrity and the progression of atherosclerotic plaques (Fig. 1). In this context, elements of metabolic dysregulation, including diabetes mellitus and an abnormal lipid profile, have been shown to trigger endothelial dysfunction. In addition, tobacco use and hypertension have been shown to accelerate luminal narrowing by increasing mechanical and oxidative stress on the vessel wall (16). While extant literature (3) emphasizes that beyond these well-established factors, newly defined risk components such as hyperhomocysteinemia, metabolic syndrome (MetS), clonal hematopoiesis, physical inactivity and a highly stressful lifestyle also accompany the pathological process of atherosclerosis, the quantitative measurement of these elements remains challenging. In addition to these modifiable variables, advanced age, male sex and genetic predisposition, as reflected by family history, are fundamental non-modifiable parameters that determine biological resistance to atherosclerotic burden (1). Notably, the transition from a healthy arterial structure to a narrowed lumen, characterized by plaque accumulation, as well as the rate of this transition is directly related to the synergistic effect of existing risk factors. Consequently, the efficacy of therapeutic strategies is contingent upon a comprehensive analysis of this multifactorial spectrum, ranging from genetic inheritance to lifestyle, and on patient-specific optimized clinical management, rather than focusing solely on a single risk factor (28). Multifactorial models are being established in a laboratory setting to thoroughly investigate the synergistic effects of these clinical risk factors on plaque development, and often use transgenic rodents and specialized dietary protocols. The combination of genetic modifications with metabolic and environmental triggers forms the foundation for constructing experimental models that accurately mimic the complex atherosclerotic process observed in the clinical setting.

FH, characterized by high LDL levels, is the most prevalent genetic disorder affecting lipid metabolism and, among hereditary conditions, exhibits a well-established association with CVD; this is attributable to dysfunction of mechanisms that eliminate LDL from plasma. Mutations in genes that encode LDLr, ApoB and PCSK9 contribute to the pathogenesis of FH (29). Notably, familial history is the major independent risk factor for atherosclerosis (28). According to the 2018 American Heart Association/American College of Cardiology Cholesterol Guideline, a family history of early atherosclerotic CVD (ASCVD) is defined as the presence of a male first-degree relative diagnosed with ASCVD before the age of 55 years or a female first-degree relative diagnosed before the age of 65 years (30). The frequency and incidence of CVD increase with age; aging at the cellular level has been demonstrated to enhance atherogenesis and facilitate plaque rupture (31). In premenopausal women, estrogen has been shown to possess anti-atherosclerotic properties; however, these properties diminish with decreasing estrogen levels post-menopause, which has been linked to an elevated risk of CVD in aging women (32). To experimentally simulate these clinical conditions, researchers primarily utilize LDLr^-/-^ and ApoB-mutant mice (simulating FH and impaired LDL clearance). These mice closely mimic the impaired clearance of LDL observed in patients with FH and develop severe atherosclerosis even on a standard diet (23,24). Furthermore, the age-related sensitivity and heightened risk of plaque rupture, as outlined in clinical guidelines, are commonly modeled in laboratory settings employing aged animal cohorts (simulating immunosenescence and progressive arterial wall deterioration) to investigate plaque instability (33).

Hyperlipidemia refers to an imbalance in cholesterol levels, namely LDL and high-density lipoprotein (HDL) cholesterol, in the bloodstream. LDL and HDL modulate cholesterol levels in the body, and the imbalance in this modulation may elevate the risk of cardiovascular problems (34). The primary factor initiating atherogenesis is the accumulation of LDL cholesterol and ApoB-containing lipoproteins within the arterial wall. Elevated LDL cholesterol has been demonstrated to be associated with ASCVD (35,36). In addition, elevated blood pressure can lead to raised shear stress on the endothelium surface, vascular remodeling and increased vascular stiffness (16), and hypertension induces atherosclerosis in large and medium-sized arteries (37). Furthermore, smoking contributes to the advancement of atherogenesis by diminishing the bioavailability of NO and inducing oxidative stress, thus amplifying the inflammatory response in the vascular endothelium (9). Modifying the adhesion and aggregation of platelets, primarily triggered by the exposure of subendothelial collagen and the subsequent release of von Willebrand factor following endothelial injury, directly influences atherogenesis (38). These activated platelets not only form microthrombi but also secrete various chemokines and growth factors that recruit leukocytes to the lesion site and promote smooth muscle cell proliferation, thereby accelerating plaque growth and destabilization. Diabetes mellitus has been shown to be associated with accelerated progression of atherogenesis. The hyperglycemic state, which is characteristic of diabetes, has been shown to create a risk for atherosclerosis by triggering dyslipidemia, increased advanced glycation end products, increased oxidative stress and inflammation (39). Systemic and local inflammation leads to the stimulation of immune system cells, particularly monocyte-derived macrophages, T lymphocytes and dendritic cells-, which contribute to endothelial dysfunction and enhance vascular permeability via the mediators they release (40,41). Experimental studies (23-25) have simulated these metabolic risk factors in genetically modified rodent models, primarily ApoE-deficient mice, and in rabbit and zebrafish models. These conditions are typically induced through specific dietary protocols, such as high-cholesterol, Western-type or high-fructose diets, which emulate clinical hyperlipidemia and insulin resistance. Furthermore, the inflammatory response and increased vascular permeability are examined by monitoring macrophage migration and the release of mediators, such as pro-inflammatory proteins (for example, C-reactive protein and interleukin-1β) and oxidative stress markers (including malondialdehyde and 8-isoprostane). These laboratory settings, which also evaluate histological changes such as aortic wall thickening, strengthen the link between clinical risk factors and the construction of experimental models.

Elevated plasma levels of homocysteine, a non-essential amino acid, have been associated with CVD. Hyperhomocysteinemia disrupts endothelial cell function with respect to vascular tone, decreases NO bioavailability and induces platelet activation. In addition, it can induce oxidative stress, trigger inflammation and enhance vascular smooth muscle cell proliferation (42). MetS is defined by a cluster of metabolic abnormalities, including insulin resistance, atherogenic dyslipidemia, central obesity and hypertension; insulin resistance and obesity-related systemic oxidative stress trigger inflammatory pathways, resulting in tissue fibrosis, atherogenesis and thus CVDs (43). Clonal hematopoiesis is driven by somatic mutations in genes such as TET2 that cause the overproduction of pro-inflammatory immune cells, a process more prevalent in the elderly and strongly linked to accelerated CVD (44). A causal link exists between inflammasome activation in macrophages resulting from clonal hematopoiesis-associated mutations and the pathophysiology of CVD (45).

Lipoprotein (a) [Lp(a)] is a LDL particle that contains Apo(a) and ApoB; elevated plasma concentrations of Lp(a) have been linked to ASCVD due to its ability to traverse the endothelial barrier and adhere to the arterial wall in a manner similar to LDL (44). In addition, Lp(a) possesses a structure similar to plasminogen and can attach to the plasminogen receptor, resulting in pro-thrombotic actions (46,47). Trimethylamine N-oxide (TMAO) is a dietary metabolite produced from phosphatidylcholine and L-carnitine through the activity of the human gut microbiome. The pro-atherogenic effects of TMAO can be initiated by provoking vascular wall inflammation, stimulating the generation of reactive oxygen species and disrupting reverse cholesterol transport. TMAO may also stimulate the expression of inflammatory cytokines and adhesion molecules (44). In the last decade, increased blood TMAO levels have been recognized as a biomarker for heightened CVD risk, and numerous studies (44,48) have noted and documented a favorable association between circulating TMAO levels and the risk of CVD.

Peripheral artery disease (PAD) is characterized by stenosis or occlusion in the aorta or arteries of the extremities, and atherosclerosis is the primary etiology of PAD (49). PAD may be asymptomatic, or manifest clinically with intermittent claudication, cramps, exhaustion, necrosis, coldness, pulselessness, pallor, paresthesia, paralysis, or pain during effort or at rest (50). Ulcers, gangrene and death can occur in the course of the disease, and some limb lesions are associated with an increased risk of amputation (51).

Coronary artery disease (CAD) is a condition characterized by the obstruction of the coronary arteries, primarily due to atherosclerosis. The clinical presentation of CAD is characterized by the manifestation of stable angina pectoris and acute coronary syndrome (52), and the resulting disorder is characterized by myocardial ischemia. Notably, patients may be asymptomatic; however, the following symptoms are indicative of the condition: Angina pectoris, dyspnea, epigastric discomfort, and pain in the left or right arm, neck or jaw. Cardiogenic shock, acute heart failure, malignant arrhythmia and cardiac arrest are all potential complications for patients with CAD (2).

Ischemic stroke is a condition characterized by cerebral tissue necrosis and neurological deficits resulting from intravascular occlusion, and atherosclerotic plaques in the cerebral arteries markedly contribute to its pathogenesis (53). Hemiplegia, alterations in vision, gait or the ability to speak or understand, and abrupt, intense headaches are prevalent symptoms observed in patients following a stroke (49). Patients with stroke can remain disabled for life, or the disease can lead to death (54).

In the treatment of ASCVDs, such as PAD and ischemic heart disease, antihypertensive drugs can be used to regulate blood pressure, glucose-lowering drugs may be administered to achieve optimal glycemic control, hypolipidemic drugs can be used to keep LDL levels at <55 mg/dl, and antithrombotic drugs may be administered to reduce the risk of atherothrombosis. In addition to these treatments, the avoidance of smoking and excessive alcohol intake, a healthy diet, low-to-moderate exercise and weight control have important roles in the prevention and treatment of these diseases (55,56).

## 4. Experimental models of atherosclerosis

Atherosclerosis is a multifactorial health problem, which is influenced by fundamental cardiovascular risk factors, such as hypertension, hyperlipidemia, smoking, obesity and diabetes mellitus, as well as hereditary and inflammatory variables (for example, elevated C-reactive protein levels, and increased activity of T cells and macrophages within the lesion) (57). Experimental models in biomedical research primarily focus on the examination of physiological and pathophysiological phenomena informed by epidemiological and clinical data. Various types of biomedical investigations still necessitate the use of animal models for evaluating molecular principles (58). Atherogenesis is a protracted process that unfolds over a number of years, during which persistent risk factors drive the life-long accumulation and transformation of lipids, inflammatory cells and smooth muscle cells within the arterial wall. Due to the limitation of human studies in this area, animal experiments are frequently used to detect, evaluate and develop solutions to the problems caused by atherosclerosis, which serves a key role in the development of CVDs (59). Animal models enable the elucidation of the processes underlying the formation and progression of atherosclerosis, the identification of possible therapeutic targets, and the examination of disease progression at different stages (60). Most animal models of atherosclerosis are established by either feeding them a diet high in fat and cholesterol, or the implementation of genetic modifications that affect cholesterol metabolism (61). Various animal species have been used to study the pathophysiology and potential therapies for atherosclerotic plaques.

**Figure 2:**
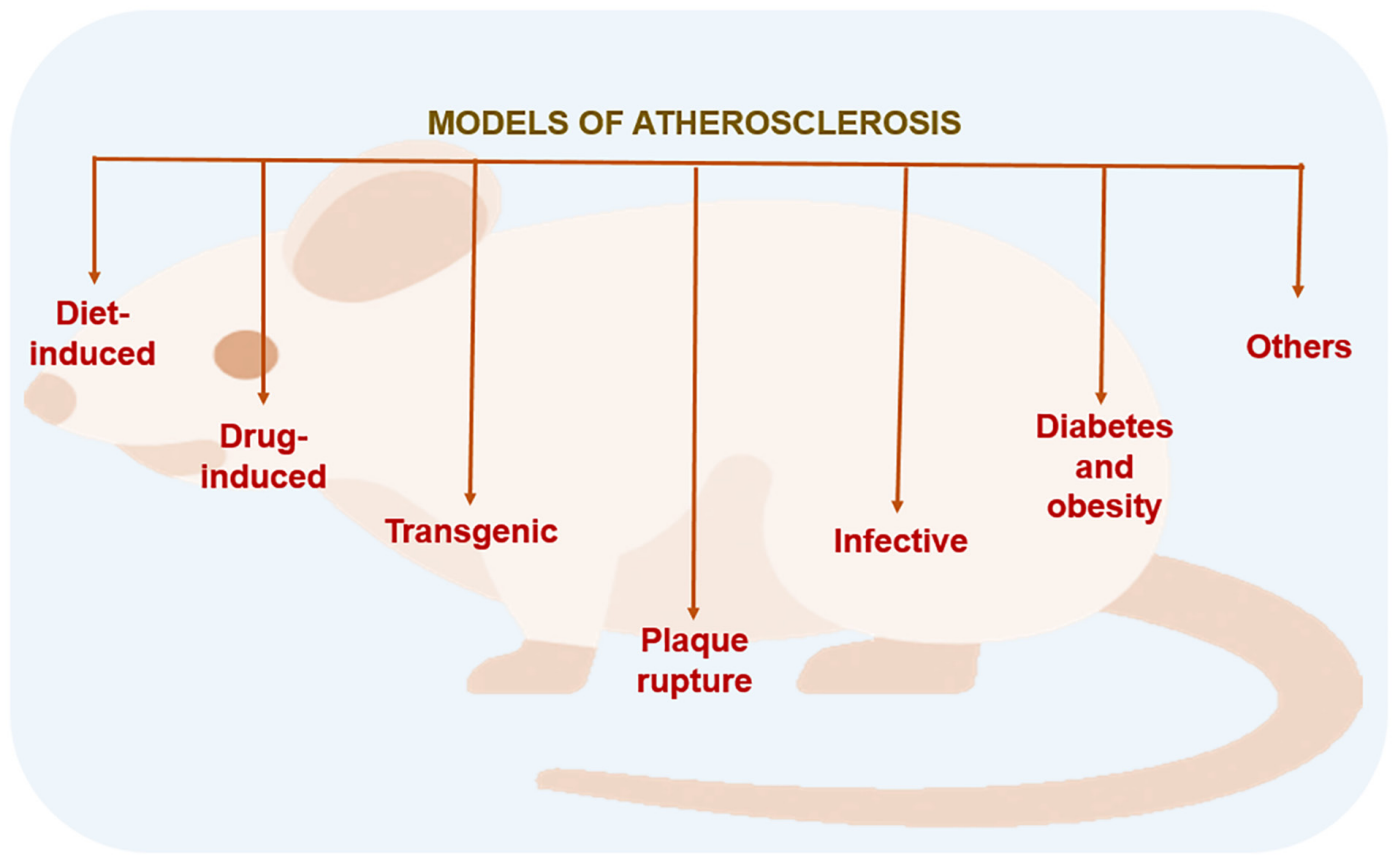
Types of models of atherosclerosis in small animals.

The first evidence for an experimental model of atherosclerosis dates back to 1908 (57,62). This model was demonstrated in the rabbit aorta; since then, experimental models have been tested in rabbits, rats, mice, non-human primates, pigs, dogs, hamsters and pigeons (44,57,60,63). As illustrated in Fig. 2, the methods employed to induce the atherosclerotic process in small animal models encompass a wide range, including diet-based, genetic, pharmacological and biological interventions. Diet-induced models provide a basis for studying hyperlipidemia and fatty streak formation, whereas transgenic models (for example, ApoE^-/-^ or LDLr^-/-^ rodents) allow for advanced plaque development by creating a background similar to human lipoprotein metabolism. In addition to these fundamental approaches, infectious agents that trigger vascular inflammation, and models of diabetes and obesity that simulate MetS are critical for analyzing interactions between atherosclerosis and comorbidities (44,60). Plaque rupture, the clinically most dangerous stage of atherosclerosis, is studied using advanced models to understand vascular instability and thrombotic events. The use of methodologies such as drug-induced and other specific approaches facilitates examination of vascular smooth muscle cell proliferation and endothelial damage in the vessel wall through focused analysis. This array of experimental models offers a controlled laboratory environment in which to assess the biological underpinnings of the multifaceted risk factors delineated in Fig. 1. In the present review, experimental atherosclerosis models in small animals have been categorized into subtopics.

### Diet-induced models

Hyperlipidemia, namely high LDL (hypercholesterolemia), is a predominant risk factor in the development of atherosclerosis and subsequent vascular disease (64). Hypercholesterolemia can be induced in animals by administering diets with varying cholesterol contents; these diets encompass a range of commercial foods that differ in their cholesterol supplementation levels, as well as in alterations to the amounts of lipids, carbohydrates, and diverse fat sources and contents, with and without the inclusion of cholic acid (65). The ingestion of saturated fatty acids has been demonstrated to elevate LDL and very LDL (VLDL) levels in humans (66). The use of rodent animal models has proven instrumental in the elucidation of the mechanisms underlying diseases associated with altered lipid metabolism (67). Mice and rats share notable genomic similarity with humans, including 30,000 protein-coding genes. In addition, the primary advantages of these experimental animal species are their brief reproductive cycles and low housing expenses (68). Furthermore, atherosclerotic lesions in rabbits can be readily induced by feeding them a high-cholesterol diet. In comparison to other animals, particularly rodents, laboratory rabbits display greater sensitivity to a high-cholesterol diet and hypercholesterolemia can be swiftly improved (69). After the first known atherosclerosis model was established in rabbits using a diet enriched in animal protein, researchers have since attempted to induce the model with diets containing different amounts of fats, cholesterol and cholic acid. The first model of atherosclerosis created in mice used a diet containing 30% fat, 5% cholesterol and 2% cholic acid; however, this was considered severe because it caused weight loss and respiratory infections (63). In a study of C57BL/6 mice in which the fat content was reduced to 15%, foam cells in aortic tissue were observed to develop at 14 weeks; notably, lesions were identified solely at the aortic root, with no lesions over the fatty streak stage reported (61). Consequently, this model is not considered suitable for investigating advanced atherosclerosis or myocardial infarction (MI), as it fails to develop complex plaques or coronary artery lesions resembling human disease.

Feeding rodents a Western diet (WD), which mimics the high-fat diet typical of Western populations, can induce experimental hypercholesterolemia and atherosclerosis (63). The WD is characterized by elevated concentrations of saturated fats (such as cocoa butter, palm oil and butter), and substantial amounts of cholesterol and cholic acid. A previous study (61) demonstrated that following this diet for 12 weeks in C57BL/6 mice can increase total and LDL cholesterol levels; this, in turn, leads to atherosclerotic changes in the aortic tissue. In addition, hypercholesterolemia has been observed in the plasma of rats fed a diet containing 4% cholesterol, 1% cholic acid and 0.5% 2-thiouracil, and atherosclerotic changes have also been detected in rats receiving this diet for 12 weeks. Notably, cholesterol ester transfer protein (CETP) expression is very low in rodents, whereas its expression is higher in rabbits; therefore, the use of rabbits in the development of atherosclerosis is more ideal for achieving hypercholesterolemia (70).

In a previous study, rats chronically fed a diet high in fructose have been demonstrated to exhibit dyslipidemia, hyperglycemia and endothelial dysfunction (71). Another study indicated that a diet high in fat and fructose can result in an increased number of foam cells and greater aortic wall thickness in Sprague-Dawley (SD) rats; a notable positive association has been observed between this dietary regimen and the onset of early atherosclerosis. The rats in this study were fed a diet consisting of 52% fat, 22% protein, 29% carbohydrates and 30% fructose solution, referred to as a high-fat, high-fructose diet (72).

Furthermore, a novel model of atherosclerosis progression, identified in Wistar rats and induced by a modified Paigen diet, has recently contributed to the literature. This modified diet is characterized by high levels of cholesterol (typically 1.25%) and cholic acid (0.5%), combined with a high-fat content (~15%) and the addition of specific sweeteners such as cocoa butter and sucrose to accelerate plaque formation. This model has been shown to simulate, in a practical, low-cost laboratory setting, cardiovascular and metabolic changes similar to those in humans with mild and severe atherosclerotic lesions by using sequential hypervitaminic and hyperlipidemic phases. These innovative experimental approaches facilitate the elucidation of specific mechanisms underlying the developmental stages of atherosclerosis and provide a solid foundation for future research (73).

### Drug-induced models

The chronic inhibition of NO synthesis induced by the administration of high doses of NG-nitro-L-arginine methyl ester (L-NAME) to rats has been shown to result in an early hypertensive state associated with vascular inflammation within 1 week and, subsequently, in severe atherosclerosis in the coronary arteries within 4-8 weeks (74). In a study by Sohn *et al* (74), a model of atherosclerosis was obtained by administering 1 mg/ml L-NAME to male SD rats weighing 170-200 g for 6 weeks. Yu *et al* (75) administered a high-fat diet and vitamin D3 to SD rats aged 8-10 weeks and observed findings related to atherosclerosis. In addition to a high-fat oral diet (15 g) once a day containing cholesterol (3%), sugar (5%), propylthiouracil (0.2%), sodium cholate (0.5%), lard (10%) and basic feed (81.3%), rats received a single dose of vitamin D3 (600,000 IU/kg) dissolved in normal saline via an intraperitoneal injection (75). Ding *et al* (76) detected foam cells in the aortic wall of C57BL/6 ApoE^-/-^ mice aged 9 weeks on a diet containing 0.3% TMAO for 8 weeks. Qin *et al* (77) fed a high-fat diet (containing 10% lard, 4% milk powder, 2% cholesterol and 0.5% sodium cholate) to male ApoE^-/-^ mice aged 8 weeks for 12 weeks, and nicotine (100 µg/ml) was also added to their drinking water for 12 weeks. At the end of the study, it was shown that nicotine increased atherosclerosis plaque size in the aortic root (77).

### Models of diabetes and obesity

It is well known that dyslipidemia and hyperglycemia can induce endothelial dysfunction, which results in vascular complications and, ultimately, atherosclerosis (78). The predominant model for studying type 1 diabetes-related atherosclerosis is the streptozotocin (STZ)-induced model in ApoE^-/-^ mice, which was first described by Park *et al* in 1998(79). A notable benefit of the STZ-induced ApoE^-/-^ murine model is the extensive body of published literature that enables researchers to compare and evaluate data, biological processes and therapeutic approaches (44,60,63,80). STZ exerts its diabetic effects by destroying pancreatic β cells. In contemporary research, this pharmaceutical compound is frequently utilized to induce a diabetic state in murine models (81). Another model of type 1 diabetes involves autoimmune destruction of pancreatic β cells following infection with lymphocytic choriomeningitis virus. Injection of this virus into LDLr^-/-^ mice can induce atherosclerosis characterized by enhanced macrophage deposits and intralesional hemorrhage, similar to that observed in humans (82). In addition to these fundamental mechanisms, STZ-induced ApoE^-/-^ mouse models serve a notable role in elucidating how diabetes accelerates the progression of atherosclerosis at the cellular and molecular levels, as demonstrated in the existing literature (79,80). A comprehensive *in vivo* study (82) demonstrated that this model, which is characterized by hyperglycemia, can increase atherosclerotic plaque formation in the aortic arch, and can exacerbate vascular smooth muscle cell proliferation and macrophage infiltration. Furthermore, STZ-induced hyperglycemia has been shown to trigger mitochondrial oxidative stress in the vascular wall and to markedly increase proinflammatory cytokine expression (such as interleukin-1β and interleukin-18) via inflammasome activation. These findings underscore the effectiveness of STZ-induced models in simulating quantitative plaque accumulation and complex pathophysiological processes involving oxidative stress, inflammasome activation and proatherogenic cellular migration. These processes form the basis of diabetic vascular complications and are consistent with human clinical findings (83).

The most common procedure for developing a model that simulates atherosclerosis in patients with type 2 diabetes or obesity involves crossbreeding ApoE^-/-^ and LDLr^-/-^ mice with mice lacking the satiety hormone leptin (ob/ob) and the leptin receptor (db/db). The satiety hormone leptin, produced by adipocytes and enterocytes, regulates appetite at the hypothalamic level. Notably, ob/ob mice possess mutations in the leptin gene, whereas db/db mice have mutations in the leptin receptor gene; thus, these mutations cause hyperphagia, leading to metabolic abnormalities. The db/db ApoE^-/-^ mice have been successfully generated, displaying traits linked to type 2 diabetes, such as weight gain, hyperglycemia, hyperinsulinemia, a three- to fourfold increase in atherosclerotic plaque load, and increased cholesterol levels by week 20 (84,85).

### Plaque rupture model

A novel genetically modified mouse model with vulnerable atherosclerotic plaques has recently been created by crossbreeding ApoE^-/-^ mice with mice possessing a heterozygous mutation (C1039^+/-^) in the Fbn1 gene. Fbn1 constitutes the principal structural element of the extracellular microfibrils within the vessel wall, and the heterozygous C1039^+/-^ mutation results in the destruction of elastic fibers (21). Notably, it has been shown that ApoE^-/-^Fbn1C1039G^+/-^ mice have a more unstable plaque phenotype than ApoE^-/-^ mice, as they experience abrupt plaque rupture, leading to MI and rapid mortality (27). In addition, previous studies (31,33) have revealed that the conventional ApoE^-/-^ model also develops a fragile plaque phenotype when subjected to a sufficiently prolonged observation period, obviating the necessity for such intricate genetic crossbreeding techniques. A previous study examined the progression of atherosclerotic plaques in ApoE^-/-^ mice; the study spanned 52 weeks (equivalent to 1 year) and utilized *in vivo* methodologies to investigate the transformation of atherosclerotic plaques. The findings revealed a gradual transition from a macrophage-dominated cellular structure to a fragile plaque phenotype characterized by an acellular, dense necrotic core and calcification. Furthermore, analyses demonstrated that despite left ventricular function remaining largely unaffected at this advanced stage, widespread and severe stenoses developed in the coronary arteries. These findings point out the relevance for the ApoE^-/-^ model for two main reasons. First, the model is instrumental in simulating the initial phase of lipid accumulation; second, it provides a framework for elucidating the pathophysiology of vulnerable plaques, which can lead to advanced clinical complications in humans (33).

### Infective models

A large body of research has shown that *Chlamydia pneumoniae* (Cpn) infection is associated with atherosclerosis. In a previous model of atherosclerosis, ApoB100only (expressing only ApoB100)/LDLr^-/-^ mice have been intranasally infected with Cpn to create a human-like lower respiratory infection. This model has yielded data demonstrating the proatherogenic effect of Cpn infection in the context of human FH (86).

A notable body of research (44,87), derived from serological and molecular biology studies, has indicated that human cytomegalovirus (CMV) infection of endothelial cells serves a pivotal role in the development of atherosclerosis. Numerous pathological and animal models (44,87) have shown that human CMV infection is involved in the etiology of coronary heart disease. Quantitative polymerase chain reaction has detected CMV in vascular endothelial cells of rabbits infected with CMV at specific time points, thereby confirming that CMV successfully infects and persists within these cells. Concurrently, CMV infection has been shown to predominantly affect the proliferation of vascular smooth muscle cells, resulting in intimal thickening. Furthermore, Du *et al* (87) reported that atherosclerosis formation was accelerated in ApoE^-/-^ herpesvirus-infected mice, and the incidence of atherosclerosis was markedly reduced with antiviral treatment.

### Transgenic models

Genetically modified animal models are utilized in preclinical experiments to more closely examine diseases that develop in humans and to investigate new treatment methods. The methods employed to achieve transgenic changes include oocyte pronuclear DNA microinjection, intracytoplasmic sperm injection, stem cell modification and somatic cell nuclear transfer. The primary strategies involve the insertion of new genes and the deletion or modification of lipid metabolism-related genes (88). Of particular relevance is the frequent use of transgenic atherosclerosis models, which are generated by altering genes associated with lipid metabolism, including ApoE, LDLr and PCSK9. Notably, in 1992, Piedrahita *et al* pioneered the establishment of the first atherosclerosis model using ApoE^-/-^ mice (89).

ApoE^-/-^ mice display increased blood cholesterol levels, a condition termed hyperlipidemia, and atherosclerosis has been detected in the aortic arch and aortic root of these mice, despite adherence to a normal diet (24). A recent development in the field is the availability of an ApoE^-/-^ rat model that exhibits characteristics analogous to those of ApoE^-/-^ mice; a notable advantage of this model is its larger size compared with mice, which enables a more thorough investigation of arteries and atherosclerotic tissues (23). Another recently developed approach involves the use of an ApoE^-/-^ rabbit model of atherosclerosis. Rabbits offer advantages over rodents in this context because their lipid profile more closely resembles that of humans. In addition, rabbits exhibit a predisposition to hyperlipidemia, even when fed a standard diet; however, a notable drawback is that an exceptionally high-cholesterol diet is necessary for the development of atherosclerosis. It has been hypothesized that this dietary regime may result in liver inflammation, which could impede the experimental process (57). To surmount the limitations of single-gene knockout models and more accurately simulate the complex MetS frequently associated with clinical atherosclerosis, researchers have focused on models combining multiple genetic modifications. Specifically, the investigation focuses on the pathophysiological interaction between atherosclerosis and type 2 diabetes, one of its major clinical triggers. To this end, double-knockout ApoE^-/-;db/db^ mouse models have been developed, combining ApoE deficiency with a leptin receptor mutation. A previous study demonstrated that this model successfully mimics the characteristics of human type 2 diabetes, including obesity, hyperglycemia, hyperglucagonemia and severe hyperlipidemia, and accelerates atherosclerosis independently of other accompanying risk factors such as hypertension. Moreover, the integration of these models offers a distinctive paradigm for the observation of isolated lipid accumulation within the vascular wall (90).

LDLr^-/-^ mice, given their lipid profiles, exhibit a high degree of similarity in structure with ApoE^-/-^ mice. A recent study (24) observed that the atherosclerotic lesion structure and plaque content of older LDLr^-/-^ mice (aged ≥6 months) resembled those of humans. A notable advantage of LDLr^-/-^ mouse models over ApoE^-/-^ models is their tendency to accumulate LDL rather than inducing hypercholesterolemia through chylomicrons, thereby aligning more closely with the human lipid profile. However, it is important to note that pharmacological agents targeting the LDLr, such as statins and PCSK9 inhibitors, are ineffective in LDLr^-/-^ mice. Consequently, studies (24,36) related to these drugs cannot be conducted using this model (24). LDLr^-/-^ rabbits also serve as a valuable model for investigating human hyperlipidemia and atherosclerosis; however, their higher cost compared with mice and lower hepatic lipase (HL) activities present notable disadvantages (91).

The ApoE/LDLr double-knockout model produces a more severe atherosclerotic phenotype in mice than the previously established single-gene knockout models. In addition, a more pronounced disease has been reported to develop in this model in the presence of a normal diet compared with in ApoE^-/-^ mice. This double-knockout model increases VLDL and LDL cholesterol levels in the bloodstream (92).

Familial dysbetalipoproteinemia is caused by mutations in ApoE, resulting in elevated levels of VLDL and chylomicrons; in this disease, the gene encoding ApoE2 is affected to a greater extent than the gene encoding ApoE3-Leiden (93). In experimental models, the targeted treatment of hyperlipidemia in ApoE2^-/-^ mice has been shown to result in a more pronounced manifestation of dyslipidemic conditions when compared with in ApoE3-Leiden^-/-^ mice (93).

New models for atherothrombotic studies have also been developed; these experimentally demonstrate advanced atherosclerotic lesions and spontaneous plaque rupture. In one model, mice with a mutant Fbn1 allele (Fbn1C1039G) have been crossed with ApoE-knockout mice, thereby producing ApoE^-/-^Fbn1C1039G^+/-^ mice. In this murine model, the Fbn1 mutation has been demonstrated to induce a breakdown of elastic fibers, resulting in increased stiffness within the arterial wall and vulnerable atherosclerotic plaques that exhibit a high propensity for rupture (94). Mutations in the Fbn1 gene have been shown to impair microfibrillar assembly, fragment elastic fibers and increase collagen deposition; these phenomena are associated with vascular aging and arterial stiffening. The ApoE^-/-^Fbn1C1039G^+/-^ mouse model is characterized by accelerated atherosclerotic plaque progression, spontaneous plaque ruptures, MI and sudden death, and provides a promising starting point for studying pharmacological interventions targeting vascular aging and CVD (95).

ApoB serves as a structural component of atherogenic lipoproteins. ApoB48 is produced in the small intestine and is present in chylomicrons, whereas ApoB100 is formed in the liver and is included in LDL, VLDL, intermediate-density lipoprotein and Lp(a). The deposition of lipoproteins carrying ApoB underneath the endothelial layer markedly contributes to the atherosclerotic process (96). The development of transgenic rabbits that express hApoB100 was first documented in 1995. A subsequent analysis of plasma lipid concentrations revealed that total plasma cholesterol and triglyceride levels were 2-3 times higher in hApoB100 transgenic rabbits than in non-transgenic controls; however, the susceptibility of ApoB100 transgenic rabbits to atherosclerosis remains to be elucidated (97). The findings of another study revealed that LDLr^-/-^ApoB^100/100^ transgenic mice, a genetically modified strain resulting from the fusion of LDLr^-/-^ mice with the ApoB^100/100^ allele, exhibited elevated LDL cholesterol levels and a heightened vulnerability to atherosclerosis (98). Beyond quantitative increases in LDL, the current literature (25,44) emphasizes that, in the pathophysiology of atherosclerosis, not only LDL quantity but also LDL structural modification and aggregation are clinically critical therapeutic targets. Recent studies (25,96) employing LDLr^-/-^ hApoB100 transgenic mice, which can simulate hApoB100 dynamics and the FH phenotype in a manner fully consistent with clinical observations, offer information regarding this issue. In an innovative experimental study using LDLr^-/-^ hApoB100 transgenic mice fed a high-fat diet, the anti-atherosclerotic effect of the drug has been demonstrated to stem from the prevention of LDL aggregation, as supported by clinical data from patients with FH. The present data demonstrate that the aforementioned transgenic animal models provide a crucial framework for understanding atherogenesis and for the development of next-generation structural therapies aimed at preserving LDL conformation (25).

Scavenger receptor class B type 1 (SR-B1), an HDL receptor involved in cholesterol metabolism, has been identified as a potential therapeutic target. Mice lacking this receptor exhibit more dysfunctional HDL, and these mice develop atherosclerosis when subjected to a high-fat diet, despite high HDL levels. SR-B1 deficiency has been shown to induce a paradoxically atherogenic state; although plasma HDL levels are elevated, these particles are dysfunctional because their cholesteryl esters cannot be selectively taken up by the liver. Consequently, the impairment of hepatic SR-B1 activity halts the final step of reverse cholesterol transport, resulting in a pro-atherogenic environment similar to that observed in low-HDL state (99). This finding suggests that the absence of SR-B1 (resulting in dysfunctional, cholesterol-rich HDL) and its overactivity (leading to excessive HDL depletion) both compromise effective reverse cholesterol transport, thereby accelerating plaque formation. In addition to its impact on cholesterol metabolism, SR-B1 deficiency has the potential to influence the development and function of lymphocytes, erythrocytes and platelets. This defect is responsible for the occurrence of fatty streaking and plaque formation during atherosclerosis by disrupting the lipid profile and affecting the homeostasis of various blood cells (100). In a recent study on this topic, it was observed that an atherogenic diet administered to SR-B1 C-terminal mutant/LDLR^-/-^ mice led to the development of occlusive atherosclerosis in the coronary arteries, advanced cardiac dysfunction and mortality. The testing of novel combinations of genetic mutations and specific dietary regimens (such as combining SR-B1 C-terminal mutants with LDLr deficiency under atherogenic diets) is imperative, as it may elucidate the development of atherosclerotic processes and the progression of cardiac disease in subsequent studies (101).

PCSK9 has been shown to associate with hepatic LDLrs, leading to their lysosomal degradation, and PCSK9 hyperactivity has been observed to increase LDL levels in humans and animals. A solitary injection of recombinant AAV (rAAV) producing gain-of-function mutant variants of PCSK9 has been shown to provoke atherosclerosis in murine and hamster models (102,103). Notably, in these studies, AAV infection did not elicit adverse effects in animals and no immunological response was observed after infection. At 30 days post-injection, total blood cholesterol levels in PCSK9-AAV transgene-bearing C57BL/6 mice have been shown to be 100% higher than in control animals; and these differences have been reported to continue to exist 1 year post-infection, thus corroborating the concept that a single AAV injection produces a lasting impact (104). This model is also employed to elucidate the role of complex risk factors, such as aging and the immune system, in the pathophysiology of atherosclerosis. For example, a recent study compared young and aged mice following identical rAAV8-D377Y-mPCSK9 injections in both groups, demonstrating that immunosenescence (the aging of the immune system) accelerates disease progression *in vivo*. Using this model, it was determined that advanced age increases plaque development independently of total cholesterol levels and drives lesions toward a more advanced phenotype characterized by increased collagen content. In addition, pro-inflammatory cells, senescent T cells and age-related B cells can accumulate in the atherosclerotic aortas of aged mice. This evidence substantiates the notion that cellular senescence and pro-atherogenic immunological changes are pivotal mechanisms that expedite atherogenesis (105).

Lp(a) is a lipoprotein involved in cholesterol metabolism, comprising an LDL-like particle and Apo(a); notably, the transgenic overexpression of the human Apo(a) gene serves as an experimental molecular target for investigating atherosclerosis. In 1992, Lawn *et al* (106) documented the first creation of Apo(a)-transgenic mice. The development of fatty streak lesions in the aortic sinus of these animals has been shown to be markedly elevated compared with in non-transgenic controls when subjected to an atherogenic diet (107). Kitajima *et al* (108) demonstrated that Lp(a) can promote the development of coronary atherosclerotic lesions in Apo(a) transgenic Watanabe hereditary hyperlipidemic (WHHL) rabbits. Furthermore, increased lesion size was shown to be associated with a higher incidence of chronic MI; it was thus predicted that Lp(a) could serve as a viable target for the treatment of hyper-Lp(a)-emia and MI (108).

CETP is a glycoprotein that is synthesized in the liver, the primary function of which in lipid metabolism is the exchange of cholesterol esters between lipoproteins; its atherogenicity remains a subject of considerable debate. It has been identified in humans, rabbits, primates, chickens and hamsters. Notably, the insertion of the CETP gene into CETP-deficient rodents has been shown to decrease HDL levels and promote atherosclerosis. Rabbits exhibit elevated CETP levels, rendering them particularly vulnerable to atherosclerosis when fed a high-fat diet (109). Research has demonstrated that the inhibition of CETP in rabbits afflicted with atherosclerosis results in a substantial reduction of atherosclerotic lesions (109).

HL is an enzyme that serves a pivotal role in the breakdown of triglycerides and phospholipids in lipid metabolism, which has been demonstrated to possess both proatherogenic and antiatherogenic properties. Although the liver synthesizes HL, macrophages at the site of atherosclerosis also secrete it. A previous study (110) observed a positive association between higher HL expression and higher LDL levels. Conversely, studies (71,110) have demonstrated that decreased expression of HL results in increased HDL levels in both humans and rodents. In ApoE^-/-^HL^-/-^ mice, larger atherosclerotic structures have been observed in comparison to ApoE^-/-^ mice alone, and the HL enzyme is absent in rabbits in contrast to its presence in humans. This scenario presents a notable challenge for the utilization of rabbits as experimental models in the context of atherosclerosis development (92,110).

Inducible degrader of the LDLr (IDOL) is an enzyme that facilitates the ubiquitination and degradation of LDLr, which has been examined in rabbits using CRISPR/Cas9. A previous study aimed to examine the function of IDOL in a species exhibiting regulatory similarities to humans, since rabbits have a similar lipoprotein profile to that of humans. The findings indicated that IDOL is prevalent in the livers of rabbits and non-human primates; however, it is scarce in mouse livers. Activation of the liver X receptor in rabbits raised hepatic IDOL mRNA levels and reduced LDLr protein abundance. By contrast, IDOL deficiency elevated levels of LDL, ApoE2 and VLDLrs in the liver, resulting in substantial decreases in atherosclerotic lesion size in both the aorta and coronary arteries. This indicates an association among IDOL, lipid metabolism and atherosclerosis in rabbits (111).

### Other models

The WHHL rabbit is an experimental model that exhibits FH. Rabbits exhibiting spontaneous coronary atherosclerosis are classified as WHHL-coronary atherosclerosis, whereas those displaying spontaneous coronary atherosclerosis accompanied by MI are termed WHHL-MI. These rabbits have notably contributed to the discovery and validation of therapeutic agents (such as statins and PCSK9 inhibitors) for hypercholesterolemia and atherosclerosis (35,112). The rabbit strains were produced at Kobe University (Kobe, Japan) and their breeding program finished in June 2018 due to the formal conclusion of the long-term project by the original developers; however, these strains remain globally accessible through the National Bio-Resource Project and other research institutions (112).

WHHL and St. Thomas' Hospital (STH) rabbits exhibit congenital defects in cholesterol metabolism. WHHL rabbits model human FH, whereas STH rabbits model human hypertriglyceridemia and combined hyperlipidemia. The hypercholesterolemic state induced in these rabbits is associated with the development of atherosclerotic lesions in the aortic arch and thoracic aorta, rather than the abdominal aorta, which is consistent with the human condition (113).

In addition to WHHL rabbits, New Zealand white (NZW) rabbits and ApoE^-/-^ rabbits can be utilized to model atherosclerosis; however, it should be noted that WHHL rabbits and ApoE^-/-^ rabbits incur markedly higher expenses compared with NZW rabbits. This cost discrepancy is primarily due to the high maintenance requirements of specialized breeding programs, the licensing fees associated with genetically modified strains and the specialized veterinary care needed for animal models with spontaneous clinical complications. WHHL rabbits present notable challenges in terms of handling and management. Furthermore, ApoE^-/-^ rabbits are not readily available in some countries and are very expensive. It is evident that NZW rabbits constitute a more viable option in terms of care, management, and cost when compared with WHHL and ApoE^-/-^ rabbits. In a study by Abd Rahim *et al*, it was demonstrated that a diet of 50 g/kg cholesterol, at a concentration of 1%, over a period of 4-8 weeks, resulted in the induction and progression of atherosclerosis in NZW rabbits (114).

Comparisons between wild-type mice and humans have revealed notable parallels in immune system activation and molecular mechanisms. However, due to their marked resistance to the development of atherosclerosis, establishing a model based on a high-fat diet remains challenging. Therefore, using ApoE^-/-^ and LDLr^-/-^ knockout and transgenic wild-type mice is recommended to establish atherosclerotic experimental models. This facilitates novel research focused on immune systems resembling those observed in humans (115).

In order to bridge the gap between experimental findings and clinical applications, recent models have been developed that emphasize reducing procedural morbidity while maintaining the complexity of human-like lesions. A notable enhancement in this domain is the transition from open surgical denudation (an invasive method used to mechanically injure the arterial wall to initiate lesion formation) to percutaneous, minimally invasive techniques. For example, Ebert *et al* (116) demonstrated that stent-retriever-mediated endothelial injury via the auricular artery in rabbits effectively induces lumen-narrowing atherosclerosis without the systemic stress of conventional surgery. Beyond procedural safety, the high reproducibility of this model provides a standardized substrate for calibrating advanced vessel wall imaging tools, such as high-field magnetic resonance imaging, optical coherence tomography and intravascular ultrasound. The resulting synergy between clinical-grade induction and multimodal imaging enables precise quantification of plaque burden and inflammation, directly aligning experimental outcomes with clinical diagnostic standards (116).

Future goals entail advancing from single-layer molecular analysis to multi-omics integration for the comprehensive mapping of the intricate vascular system. Mitić *et al* (117) emphasized that the integrated use of genomics, transcriptomics, proteomics and metabolomics is crucial for pinpointing the regulatory centers of plaque stability. Emerging technologies, such as single-cell RNA sequencing and spatial transcriptomics, enable the identification of distinct cellular phenotypes inside the vascular wall with unparalleled precision. When utilized in optimized animal models (including larger species such as minipigs or transgenic swine that better mimic human arterial anatomy), these technologies can discern novel biomarkers of plaque vulnerability and biochemical signs of neointimal formation, facilitating the creation of tailored therapeutic approaches for peripheral and coronary artery illnesses (117).

The experimental approaches used to systematically investigate the multifactorial nature of atherosclerosis have varying advantages and limitations depending on the specific research focus (Table I). Dietary models in rodents are economical for studying early-stage, lifestyle-related lesions; however, they may be inadequate for modeling complex plaque formation due to factors such as low CETP expression. By contrast, rabbits are a more suitable model for this purpose due to their cholesterol-sensitive physiology and human-like lipid profiles (94). ApoE^-/-^ and LDLr^-/-^ mice, as well as ApoB100 or Apo(a) transgenic rabbits, have been used in genetic studies and provide highly reproducible lesions. However, these models rely on genetic manipulation rather than the natural course of the disease, which is a limitation that must be considered. Notably, L-NAME for hypertension, vitamin D3 for vascular calcification, and TMAO or nicotine applications successfully simulate specific clinical presentations when used to isolate risk factors. STZ-induced models of diabetes and leptin mutations, such as ob/ob or db/db, are specialized tools for understanding the effects of MetS on the vascular wall (80,118). Although infectious approaches, such as those involving Cpn, herpes or CMV, offer the opportunity to examine the immune response when testing the triggering role of inflammation, it should be noted that these methods cannot fully replicate the chronic, decades-long process observed in humans. Consequently, given translational barriers such as HDL dominance and the absence of spontaneous plaque rupture in rodents, methods that are closer to clinical standards, such as the stent retriever applied in WHHL or NZW rabbits, are critical for enhancing the predictive capacity of findings regarding human clinical outcomes (33,116). A prime example of this is the use of percutaneous stent-retriever-mediated endothelial injury in rabbit models (such as WHHL or NZW rabbits). In contrast to conventional open surgical denudation, this minimally invasive technique involves the introduction of a stent-retriever via the auricular artery to induce controlled endothelial damage mechanically. This process instigates a localized fibro-proliferative response and accelerates the formation of complex, lumen-narrowing atherosclerotic lesions that closely resemble the morphology of advanced human plaques. Consequently, this markedly reduces procedural morbidity and systemic stress for the animal.

A comparison of these experimental platforms reveals their distinct advantages and limitations for specific research purposes. Diet-induced models are cost-effective and mimic lifestyle-related early atherogenesis; however, they generally fail to reproduce complex lesions (119). Conversely, transgenic models (such as ApoE^-/-^ and LDLr^-/-^) are well-suited for molecular mechanistic studies, and have been shown to produce reproducible and advanced plaque formations. However, these models depend on genetic manipulations that do not fully reflect the natural etiology of the disease. To address the multifactorial nature of human pathology, researchers have increasingly utilized combined approaches, such as incorporating a high-fat diet or chemical induction in transgenic or diabetic backgrounds. While these dual or triple models accelerate plaque instability, a notable translational gap remains between animal models and human clinical practice (60). Rodents exhibit fundamental differences in lipid metabolism (characterized by HDL dominance) and in hemodynamic profiles compared with humans (94). Furthermore, spontaneous plaque rupture, which is the primary cause of human MI, rarely occurs in rodents (120). Consequently, the selection of a model should be meticulously aligned with the particular clinical inquiry, recognizing that preclinical findings may not invariably predict human outcomes. Table I summarizes the relative merits, drawbacks and key elements of the aforementioned atherosclerosis models.

## 5. Conclusion

Despite their inability to fully replicate human pathology, animal experimental models remain indispensable tools for elucidating the biochemical and molecular pathways of atherosclerosis. Transgenic models have enhanced the comprehension of genetic mechanisms; however, subsequent research must concentrate on closing the translational gap. The integration of novel technologies may provide a promising option to overcome the limitations of traditional animal models. Additionally, the optimization of technological models to more accurately reflect human comorbidities will enhance their clinical relevance. Consequently, the transition from descriptive studies to studies incorporating these advanced methods may accelerate the discovery of novel therapeutic targets for ischemic diseases.

## Figures and Tables

**Table I:** Characteristics of models of atherosclerosis in small animals.

Model and species	Advantages	Disadvantages	Materials and methods	(Refs.)
Diet-induced models				
Rats and mice	Similar genomic structure to humans; brief reproductive cycle; low housing expenses	Lower CETP expression vs. rabbits	High-fat diet; diets supplemented with pro-atherogenic agents, including cholesterol, cholic acid, 2-thiouracil, and/or fructose; Western diet; Paigen diet	(61,64-73)
Rabbits	Increased sensitivity to high cholesterol; higher CETP expression vs. rodents	Low hepatic lipase activity	High-cholesterol diet; high-fat diet	(69,70,91)
Drug-induced models				
Rats and mice	Low costs, easy care and short breeding cycles	Some medicines are costly and difficult to obtain	L-NAME; vitamin D3; propylthiouracil; sodium cholate; trimethylamine N-oxide; nicotine	(74,75)
Diabetes and obesity models				
Rats and mice	Increased macrophage accumulation and intra-lesional hemorrhage similar to that seen in humans; similar characteristics of diabetes in humans	Unfit for models of spontaneous plaque rupture and thrombosis	Destroying pancreatic β cells (diabetes induced with STZ or infection with the lymphocytic choriomeningitis virus); mutations in leptin genes (ob/ob) and leptin receptor genes (db/db); STZ-induced ApoE^-/-^ mice	(80,82-85)
Plaque rupture model				
Mice	Mimics complete occlusion with the presence of more unstable plaques	High and rapid mortality rates in mice	ApoE^-/-^; Fbn1C1039G^+/-^	(27)
Infective models				
Mice	Similar genomic structure to humans; low housing expenses	A costly and complex experimentation process	Intranasal administration of *Chlamydia pneumoniae*; infection with herpesvirus	(86,87)
Rabbits	Observed proliferation of vascular smooth muscle cells and intimal thickening in atherosclerotic plaques	Difficulty of care and high costs	Infection with cytomegalovirus	(87,114)
Transgenic models				
Rats, hamsters and mice	Observed lesion development in the aorta even with a normal diet; atherosclerotic tissues can be examined more comprehensively due to rats being larger in size than mice; similarity of atherosclerotic lesion structure and plaque content to that in humans; more severe atherosclerosis with double knockout (compared with either single-mutant models alone); no spontaneous plaque rupture and thrombosis observed in double knockout mice; able to conduct studies involving pharmacological interventions targeting vascular aging and CVD-related conditions in ApoE^-/-^Fbn1C1039G^+/-^ mice; changes in the homeostasis of various blood cells in addition to hyperlipidemia in SR-B1^-/-^ mice; observation of larger atherosclerotic structures in ApoE^-/-^/HL^-/-^ mice vs. ApoE^-/-^ mice	Inappropriateness of LDLr^-/-^ mice due to mechanism of action in studies using PCSK9 inhibitors and statins (since these agents require functional LDLr to exert their lipid-lowering effects); risk of MI and sudden death in ApoE^-/-^Fbn1C1039G^+/-^ mice	ApoE^-/-^ mice; ApoE^-/-^ rats ; LDLr^-/-^ mice; ApoE^-/-^/LDLr^-/-^ mice; ApoE2^-/-^ mice; ApoE3-Leiden^-/-^ mice; ApoE^-/-^ Fbn1C1039G^+/-^ mice; LDLr^-/-^/ApoB^(100/100)^ mice; SR-B1^-/-^ mice; SR-B1 C-terminal mutant/LDLr^-/-^ PCSK9-AAV mice; PCSK9-AAV hamsters; Apo(a) transgenic mice; CETP transgenic rodents; ApoE^-/-^/HL^-/-^ mice; ApoE^-/-^; db/db mice; humanized ApoB100 LDLr-knockout mice	(23-25,90,92-95,98-104,109-111)
Rabbits	Human-like lipid profiles; tendency to hyperlipidemia even with standard diet; observation of a marked increase in cholesterol and triglycerides in ApoB100 transgenic rabbits; presence of coronary atherosclerotic lesions and similarity to chronic MI in Apo(a) transgenic WHHL rabbits; regression of atherosclerosis by CETP inhibition	Dietary requirement with very high cholesterol levels for the development of atherosclerosis; risk of liver damage due to the need for a high cholesterol diet; low HL activity	ApoE^-/-^ rabbits; LDLr^-/-^ rabbits; ApoB100 transgenic rabbits; Apo(a) transgenic; WHHL rabbits; CETP transgenic rabbits	(91,92,97,108-110)
Other models				
Mice	Similarity to humans in terms of molecular mechanisms of the immune system	Presence of small anatomical structures for atherosclerotic tissue examination; resistance to the development of atherosclerosis	Genetically modified strains (ApoE^-/-^ or LDLr^-/-^ mice) and wild-type mice (specifically C57BL/6 background, requiring environmental or dietary induction protocols such as thermoneutral housing or Western diet)	(24,115)
WHHL and STH rabbits	Observation of spontaneous coronary atherosclerotic and MI lesions; development of atherosclerosis in aortic arch and thoracic aorta	Not developing of atherosclerosis in abdominal aorta; high expenses; difficulty of use and management	WHHL and STH rabbits	(111-113)
NZW rabbits	More viable option in terms of care, management and cost vs. WHHL rabbits	Difficulty of care and high costs; low HL activity	High-cholesterol diet feeding or stent-retriever-mediated endothelial injury	(91,114,116)

AAV, adeno-associated virus; Apo, apolipoprotein; CETP, cholesterol ester transfer protein; CVD, cardiovascular disease; Fbn1, fibrillin-1; HL, hepatic lipase; MI, myocardial infarction; LDLr, low-density lipoprotein receptor; NZW, New Zealand white; PCSK9, proprotein convertase subtilisin/kexin type 9; SR-B1, scavenger receptor class B type 1; STH, St. Thomas' Hospital; STZ, streptozotocin; WHHL, Watanabe hereditary hyperlipidemic.

## Data Availability

Not applicable.
